# Beyond orthography: a refined perceptual assimilation task paradigm for measuring cross-linguistic phonetic similarity

**DOI:** 10.3389/fpsyg.2026.1859283

**Published:** 2026-07-23

**Authors:** Jing Yang, Yufeng Chen

**Affiliations:** 1 International College, Dongguan University of Technology, Dongguan, China; 2 School of Literature, Shandong University, Jinan, China

**Keywords:** cross-linguistic similarity, logographic background, orthographic independence, perceptual assimilation task, sibilants, speech acquisition

## Abstract

To address the limitations of traditional Perceptual Assimilation Task paradigms—specifically their heavy reliance on phonetic notation and metalinguistic knowledge—this study proposes a refined, orthography-independent paradigm. Designed to enhance ecological validity, the new framework uses auditory, syllable-based stimuli to decouple speech perception from orthographic interference. This approach is particularly suited for native Chinese speakers from logographic backgrounds who may lack proficiency in IPA. Applying this paradigm to the perception of Mandarin sibilants by Cantonese speakers, our results corroborate established dialectological observations, demonstrating that participants are sensitive to fine-grained phonetic variation across different vocalic environments. By providing more granular similarity data, this methodology offers a robust, versatile, and generalizable tool for measuring cross-linguistic phonetic similarity across diverse non-alphabetic language contexts and predicting second-language speech acquisition outcomes.

## 1 Introduction

In the field of second language (L2) speech acquisition, dominant theoretical frameworks regard cross-language phonetic similarity as a critical factor for predicting and explaining L2 acquisition performance. Key examples include the Speech Learning Model and its revision (SLM/SLM-r; Flege et al., 2021), the Perceptual Assimilation Model for L2 learners (PAM-L2; Best and Tyler, 2007), and the Second Language Linguistic Perception model (L2LP; Escudero and Yazawa, 2024). To determine cross-language phonetic similarity, Strange and Shafer (2008, p. 172) summarized four techniques commonly employed in recent L2 speech perception research: (1) qualitative descriptions of articulatory-phonetic similarities; (2) qualitative perceptual comparisons involving the (narrow) transcription of L2 segments; (3) acoustic comparisons of native language (L1) and L2 phones, including the use of correlational techniques such as discriminant analysis; and (4) direct measures of perceived/perceptual similarity, which involve presenting L2 segments for listeners to categorize in terms of L1 phonetic categories. However, recent research suggests that direct measures of perceptual similarity are not always predictable from acoustic comparisons (cf. Strange et al., 2004, 2005). Thus, direct perceptual measures represent a more valid approach for determining L1/L2 perceived similarities for both naïve listeners and L2 learners.

### 1.1 Measurement paradigms for cross-linguistic speech perceptual similarity

Rochet (1995), Bohn (2002), and Strange and Shafer (2008, p. 54) argue that learners’ perceptual assimilation patterns provide the most accurate reflection of cross-language phonetic similarity. The associated experimental methodology is known as the Perceptual Assimilation Task (PAT). The PAT has been extensively utilized in numerous studies (e.g., Bohn and Flege, 1992; Polka and Bohn, 1996; Guion et al., 2000; Best et al., 2001; Best and McRoberts, 2003; Strange et al., 2004, 2005; Bohn and Best, 2012). While specific implementation procedures vary across the literature, three primary paradigms have emerged:

The first paradigm requires participants to provide a native orthographic symbol for each non-native stimulus, thereby yielding information regarding the mapping between phonetic stimuli and L1 phonemic categories.

The second paradigm involves participants providing graded similarity ratings for the perceived distance between L1 and L2 speech sounds (Tulving, 1981). In this approach, subjects compare specific L1–L2 pairs and rate them on a scale ranging from “very similar” to “very dissimilar,” with the degree of similarity typically determined via a Likert-type scale (Fox et al., 1995; Hao, 2012; Yang et al., 2022). Compared to the first paradigm, this method yields more fine-grained data.

The third paradigm requires participants to first select the L1 category most similar to the L2 stimulus and subsequently rate the degree of similarity between the two. This strategy has been employed in multiple studies (e.g., Flege et al., 1991; Guion et al., 2000; Best and Tyler, 2007; Levy, 2009; Hao, 2012; Yang, 2020; Yang et al., 2022). Notably, some researchers (e.g., Guion et al., 2000) combine categorization and rating data into a single metric called the “fit index,” providing a weighted measure of the L1-to-L2 mapping. In Guion et al.’s (2000) study, for instance, Japanese native speakers identified the English [b] as Japanese /b/ in 84% of trials, with these mappings receiving an average goodness-of-fit rating of 5.3 on a scale from 1 (poor exemplar) to 7 (excellent exemplar). By multiplying the identification proportion (0.84) by the mean rating, a fit index of 4.5 was derived for the English /b/ to Japanese /b/ mapping.

### 1.2 Limitations of current perceptual assimilation task paradigms

However, the three aforementioned paradigms are each associated with various limitations. Regarding the first paradigm, Bohn et al. (2011) noted that when participants are permitted to select orthographic symbols freely, their responses can be difficult to interpret. To address this, fixing the response options might seem to mitigate such interpretational difficulties. Nevertheless, Park and de Jong (2008) argued that because many languages utilize the Roman alphabet, learners may conflate phonemes with orthography during the experiment. This confusion often leads to orthographic interference, where participants rely more heavily on written forms than on auditory categorization, thereby compromising the accuracy of the findings. Another modified approach involves forced-choice tasks; for instance, Wang and Deng (2009) required participants to select one category from a predefined set of native sounds upon hearing the stimulus. However, the primary drawback of these methodologies is their inability to capture the nuanced details of similarity degrees.

The primary drawback of the second paradigm lies in its impracticality; participants are required to provide similarity ratings for an extensive number of stimulus pairs, which has limited its widespread adoption in empirical research (Bohn, 2017). For instance, in the study by Flege et al. (1994), pairing three Spanish vowels with seven English vowels within a single phonetic environment resulted in 405 vowel pairs (including distractors). When considering multiple phonetic environments, the required number of pairs could easily escalate into the thousands.

While the third paradigm is currently considered the most optimal approach, it nonetheless faces challenges regarding adaptability. Park and de Jong (2008) pointed out a significant flaw: the scoring system relies heavily on the frequency with which orthographic or International Phonetic Alphabet (IPA) options are selected, rather than accounting for the underlying mechanisms or generalizable patterns behind these frequencies. A high fit index may simply reflect the frequent selection of a particular label, which does not necessarily equate to high phonetic similarity between the stimulus and the category. Other confounding factors may drive frequent selection, such as a learner’s tendency to choose options that are more familiar or easier to recall. Furthermore, a specific label might be frequently selected because it shares moderate similarity with multiple distinct stimuli, rather than having high similarity with one specific stimulus. Consequently, predictions based solely on such proportion-based calculations serve, at best, as indirect indicators of the goodness-of-fit between a stimulus and an L1 category.

In summary, despite the various limitations inherent in current PAT paradigms, there is a consensus among scholars that perceptual similarity derived via PAT is more reliable than IPA-based comparisons or acoustic analysis. Bohn (2017) maintains that these methodologies represent a significant contribution to cross-language speech perception research, demonstrating that the basis for cross-language identification is “substantial rather than formal” (Catford, 1968, p. 164). At present, the third paradigm offers the most effective balance between measurement granularity and experimental workload, although further refinement is required to address the persistent issues related to orthographic interference. Crucially, while this orthographic interference presents a significant confounding variable in studies involving alphabetic languages—where participants might conflate phonemes with written orthographic symbols—it becomes an absolute structural barrier in logographic environments where written text cannot isolate individual speech segments, and where learners often lack phonetic notation training.

### 1.3 Current research

To test the boundaries of current PAT methodologies in non-alphabetic settings, the current study addresses the difficulties encountered by Chinese dialect speakers during the acquisition of Mandarin speech. As the official language of China, Mandarin is extensively promoted throughout diverse dialectal regions. Notably, the phonological systems of certain Chinese dialects diverge so significantly from Mandarin that they are mutually unintelligible, despite sharing a unified writing system—Chinese characters (*Hanzi*). However, *Hanzi* is not a phonetic script; the phonetic information carried by a single character exceeds the scope of a single segment. Consequently, *Hanzi* cannot serve as response options for segmental similarity measurements in a PAT. Furthermore, the vast majority of dialect speakers have received no formal training in Pinyin or the International Phonetic Alphabet (IPA). This unique intersection of a logographic background and a lack of formal metalinguistic notation creates a methodological deadlock: traditional text-based PAT paradigms cannot be validly applied. Given these constraints, the present study proposes an enhancement to the existing PAT paradigms by eliminating text entirely and introducing a completely audible, syllable-based alternative.

The specific research question focuses on the acquisition of Standard Mandarin sibilants by native Cantonese speakers. Previous studies have indicated that Cantonese speakers encounter significant difficulties in both the perception and production of Mandarin sibilants, primarily manifesting as the confusion of different places of articulation (Wang, 1951; Li et al., 1988; Wu, 2001; Hu, 2016). A brief overview of the Mandarin and Cantonese sibilant systems is provided here. Mandarin sibilants are categorized into three sets based on their place of articulation: dental-alveolars ([ts, tsʰ, s]), post-alveolars ([tʂ, tʂʰ, ʂ]), and alveolo-palatals ([ʨ, ʨʰ, ɕ]), with the former two sets conventionally co-referred to as apical sibilants. Lee and Zee (2010) provide more detailed transcriptions and descriptions: [ts, tsʰ, s] are characterized as apico-laminal or laminal denti-alveolar; [tʂ, tʂʰ, ʂ] as apical post-alveolar; and [ʨ, ʨʰ, ɕ] as apico-anterodorsal or lamino-anterodorsal alveolo-palatal. Conversely, Standard Cantonese possesses only a single set of laminal alveolars ([tʃ, tʃʰ, ʃ]; Zee, 1999), which remains phonetically distinct from all three Mandarin series in terms of fine-grained articulatory configurations.

Crucially, the vocalic environment heavily conditions the phonetic realization of Cantonese sibilants; they exhibit a perceptible degree of palatalization in the context of high front vowels (Hashimoto, 1972; Bauer and Benedict, 1997; Yu, 2016) and rounded vowels (Wong, 1941; Kao, 1971; Bauer and Benedict, 1997; Cheung, 2002; Yu, 2016; Chan, 2017).^1^ Given that the phonetic realization of sibilants is strongly conditioned by the following vocalic environment, the current paradigm presents the target sounds within natural syllable frames rather than as isolated consonants. This approach allows for a more nuanced baseline, considering that Cantonese sibilants—whether palatalized or not—merely approximate, rather than fully match, their Mandarin [ts, tsʰ, s] or [ʨ, ʨʰ, ɕ] counterparts (Li, 1994; Li et al., 1995; Bauer and Benedict, 1997; Yuan, 2001; Cheung, 2002; Zhan, 2004; Chen, 2011; Rao et al., 2020).

Because *Hanzi* cannot serve as visible response options for segment-level perceptual similarity judgments, the present study refines the third PAT paradigm described in Section 1.2 by replacing written labels with audible speech stimuli. We anticipate that the efficacy of this refined paradigm will ultimately be evaluated against three core criteria. First, the empirical findings are expected to align with established dialectological knowledge. Specifically, because Cantonese learners consistently exhibit profound difficulty in differentiating Mandarin sibilant places of articulation, we hypothesize that participants will assimilate Mandarin sibilants with different places of articulation into a single Cantonese category sharing the same manner of articulation (e.g., mapping [s], [ʂ], or [ɕ] onto Cantonese [ʃ]), while cross-manner assimilations (e.g., [ts] to [ʃ]) should be extremely rare.

Second, the paradigm is designed to capture cross-linguistic phonetic similarity variations conditioned by differing phonological environments. Drawing upon prior literature, we hypothesize that participants will assign higher similarity ratings between Mandarin alveolo-palatals and Cantonese laminals in rounded vowel environments compared to unrounded ones, due to palatalization in rounded Cantonese vowel contexts.

Third, the refined approach needs to demonstrate robust generalizability across distinct sound classes, expanding its scope to complex phonetic contrasts involving aspiration and manner of articulation (i.e., affricates and fricatives). Given the necessity of presenting these obstruents within natural syllabic contexts (as noted above), the precise operationalization of these criteria is delineated below, followed by an evaluation of the approach’s feasibility and theoretical value.

## 2 Materials and equipment

### 2.1 Stimuli design

Real words were selected as experimental stimuli, as shown in Table 1. The Mandarin stimuli comprised 18 syllables, consisting of the target consonants ([ts, tsʰ, s], [tʂ, tʂʰ, ʂ], and [ʨ, ʨʰ, ɕ]) combined with the rimes /an/ and /uan/. The Cantonese stimuli included 10 syllables, featuring the target consonants ([tʃ, tʃʰ, ʃ]) paired with the rimes /a:n/ and /y:n/. Additionally, syllables beginning with [t] and [tʰ] were included as distractors. The design considerations are as follows:

**Table 1:** Stimuli.

Mandarin sibilants	/an/[an ian]	/uan/[uan yan]
[ts]	簪 “hairpin”	钻 “drill”
[tsʰ]	参 “refer”	蹿 “leap up”
[s]	三 “three”	酸 “acid”
[tʂ]	詹 “a common surname”	专 “devoted”
[tʂʰ]	掺 “mix”	穿 “wear”
[ʂ]	衫 “shirt”	栓 “bolt”
[ʨ]	煎 “fry”	捐 “donate”
[ʨʰ]	千 “thousand”	圈 “loop”
[ɕ]	先 “earlier”	宣 “announce”
Cantonese sibilants	/a:n/	/y:n/
[tʃ]	赞 “praise”	专 “devoted”
[tʃʰ]	餐 “meal”	穿 “wear”
[ʃ]	山 “mountain”	酸 “acid”
[t]	丹 “pill”	端 “hold”
[tʰ]	滩 “beach”	湍 “rushing waters”

All onset consonants are transcribed using the International Phonetic Alphabet (IPA). The phonetic symbols for Mandarin rimes refer to Modern Chinese (6th Revised Edition) (Huang and Liao, 2017), while those for Cantonese rimes refer to the Glossary of Standard Cantonese Pronunciations (Zhou and Rao, 1988) and the Dictionary of Standard Cantonese Pronunciation (Zhan, 2002).

Since the target consonants cannot occur in isolation, they were presented within carrier syllables for testing. To account for varying phonetic environments, we selected /an/ and /uan/ to represent unrounded and rounded contexts, respectively. However, due to phonotactic constraints, the rime inventories of Mandarin and Cantonese do not perfectly align. To minimize the influence of rime inconsistency, we selected the most similar rimes possible and ensured consistency in the phonotactic patterns between Mandarin and Cantonese syllables during stimuli selection. Based on these considerations, the Cantonese /y:n/ rime was chosen to correspond with the Mandarin /uan/ (realized as [uan] or [yan]).^2^ Furthermore, to eliminate the confounding effect of lexical tone, all syllables were produced with level tones. Syllables beginning with [t] and [tʰ] served as distractors; since these alveolar stops share a similar place of articulation with the target sibilants, they allow for a more rigorous assessment of whether Cantonese speakers exhibit assimilation patterns beyond the expected affricate and fricative categories.

### 2.2 Participants

#### 2.2.1 Speech stimuli producer

The Mandarin speech stimuli were recorded by two professional broadcasters (one male, one female). Both speakers held a National Proficiency Rating of Level 1-B and were native speakers of the Northern Mandarin dialect.

For the production of Cantonese stimuli, we first recorded spontaneous self-narratives from three native Cantonese speakers who were born and raised in the traditional old districts of Guangzhou. These audio files were then distributed to three additional native Cantonese evaluators (also from the old districts), who were tasked with selecting the speaker with the most authentic accent and an ideal phonetic profile to serve as the informant for the experiment. Based on this peer-evaluation process, a middle-aged male was ultimately selected as the Cantonese informant.

#### 2.2.2 Perception experiment participants

Forty-two native residents from the traditional historic districts of Guangzhou were recruited for this study. These individuals ranged in age from 50 to 69 years. All participants were dominant speakers of Guangzhou Cantonese with no proficiency in other dialects, and none had received formal education in *Hanyu Pinyin* (the Mandarin phonetic alphabet). Before the experiment, they reported no history of hearing impairment or speech disorders and underwent a simplified audiometric screening procedure.

### 2.3 Equipment and environment

All experimental stimuli were recorded in a professional sound-treated studio (with an average ambient noise level below 30 dB). The recording apparatus consisted of an AKG P120 professional condenser microphone, a Yamaha UR22C external sound card, and a Lenovo ThinkPad X13 laptop. Recordings were captured via xRecorder software (a customized application developed based on Praat) at a sampling rate of 44,100 Hz and a bit depth of 16-bit in mono-channel format.

The perception experiment was implemented using PsychoPy (version 2021.2.3; Peirce, 2007). Participants completed the task independently through the program’s graphical user interface (GUI) in a quiet room or a phonetics laboratory (average ambient noise < 45 dB). Subjects were seated in front of a computer while a researcher provided instructions and supervision (to record any accidental input errors in real-time). To minimize environmental noise interference, participants were required to wear closed-back headphones (AKG K92) during the experiment.

## 3 Methods

### 3.1 Stimulus selection and processing

During the recording session, informants were instructed to produce each monosyllabic item three times, with distinct intervals between tokens. Speakers were required to maintain clear articulation and a natural speaking rate. Following the recording, a doctoral student specializing in phonetics and a graduate student in linguistics (a native Cantonese speaker) were invited to perform an auditory vetting of the Mandarin and Cantonese materials, respectively. They were tasked with selecting the most natural and representative token from the three repetitions of each word (cf. Levy, 2009; Hao, 2012; Yang et al., 2022). Subsequently, the selected stimuli underwent duration normalization based on the manner of articulation and were intensity-normalized to a uniform level of 70 dB to serve as the final auditory stimuli for the Perceptual Assimilation Task.

### 3.2 Experimental design

The experiment consisted of a practice session and a formal testing session. The practice session was designed to familiarize participants with the experimental procedure and interface; it utilized CV syllables with monophthongal rimes (/a/ and /i/). The Mandarin onsets used in the practice session—stops ([t, tʰ, k, kʰ]) and fricatives ([f, x])—did not include any of the target sibilants investigated in this study. Data from the practice session were excluded from the final analysis. The stimuli for the formal experiment were those described in §2.1.

Given that the Mandarin stimuli were recorded by both a male and a female informant, including all possible trials would have doubled the experimental duration. To reduce the cognitive load on participants and minimize task fatigue, the stimuli were divided into two versions (Version A and Version B), with each version containing an equal distribution of male and female voices. A counterbalancing principle was applied: for consonants sharing the same manner of articulation but differing in place of articulation, the gender of the stimulus voice remained consistent when paired with the same rime. For instance, when [ts], [tʂ], and [ʨ] were combined with the rime /a/, all stimuli were sourced from the same informant gender within a single version.

Participants were randomly assigned to either Version A or Version B. To control for potential bias, we maintained an equal distribution of experimental versions and a balanced gender ratio. After excluding those with incomplete data, 38 participants (19 males, 19 females) were ultimately included in the final analysis.

### 3.3 Experimental procedure

Prior to the commencement of the experiment, researchers informed participants that the task involved matching Mandarin pronunciations with Cantonese counterparts. Specifically, participants were instructed to compare the onset of a Mandarin stimulus with five Cantonese response options and select the one they perceived as most similar. After the briefing, participants listened to a spontaneous speech sample from the Cantonese informant; this auditory familiarization phase helped participants establish a baseline for the informant’s specific voice characteristics. The entire experiment was conducted via mouse clicks. Participants were permitted to pause and rest at any time. On average, the experiment was completed in approximately 20 min. The specific procedure was as follows:

First, an instruction screen was presented, allowing participants ample time to read and understand the task requirements. Researchers were available to clarify any procedural questions. Upon confirming their comprehension, participants initiated the experimental program via a spacebar press—an action concurrently serving as their implied informed consent. They then proceeded to a practice session designed to familiarize them with the experimental flow and response interface under researcher guidance.

**Figure 1:**
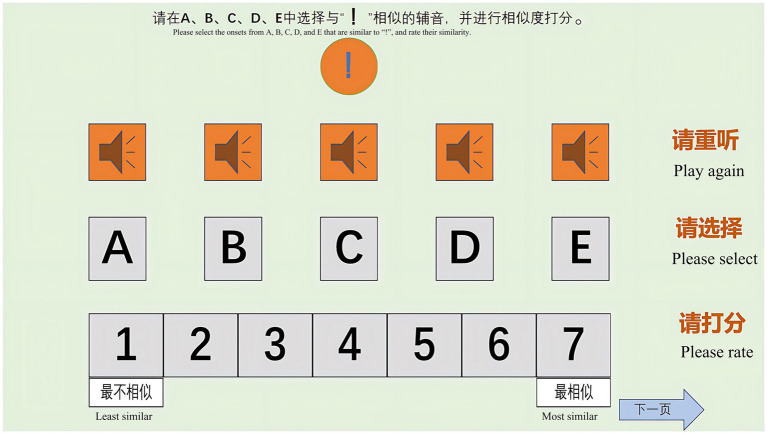
Graphical user interface of the perceptual assimilation task.

The interface for the formal experiment was identical to that of the practice session (Figure 1). In each trial, participants first clicked the “!” icon to play the Mandarin target stimulus before listening sequentially to the five Cantonese response options (labeled A–E). It was strictly mandatory for participants to listen to the target stimulus and all five response options in their entirety before making a selection. For each trial, participants identified the sound perceptually closest to the target and rated its similarity on a 7-point Likert scale (ranging from 1 to 7). To minimize memory load, the order of the five Cantonese consonants remained fixed throughout each block (e.g., [tʃ, tʃʰ, ʃ, t, tʰ] corresponding to labels A–E). Furthermore, the distractors [t, tʰ] (labels D and E), which exhibited the greatest phonetic contrast, were placed at the end to facilitate the formation of stable mental mappings (or “symbolization”). These distractors also served as a mechanism to monitor participant engagement and task compliance. Within each trial, all audio files—including the stimulus (“!”) and options (A–E)—could be replayed independently and indefinitely. This allowed participants to make informed judgments, although selections could not be altered once submitted.

The practice session consisted of eight trials, featuring four Mandarin stimuli from the male informant and four from the female informant; these trials were excluded from the formal experiment. During the practice phase, if a researcher observed a significant discrepancy between a participant’s perceptual matching or similarity ratings and the expected outcomes, they would confirm again with the participant to ensure a thorough understanding of the experimental requirements. Formal testing commenced only after participants completed the practice session and confirmed they had no further questions regarding the operation or task demands.

The formal testing phase consisted of 18 non-repeated critical trials (9 target sibilants × 2 rimes). This streamlined trial count served as a methodological trade-off, balancing data efficiency with practical field constraints, notably preventing cognitive fatigue among the older participants. The procedure for the formal experiment remained identical to the practice session, though researchers provided no further guidance or prompts. All experimental trials were presented in a randomized order. The program automatically recorded the participants’ perceptual choices (A/B/C/D/E) and their corresponding similarity ratings (1–7).

## 4 Results

The data analysis initially involved calculating assimilation proportions to provide a preliminary overview of the participants’ perceptual patterns, followed by fitting Cumulative Link Mixed Models (CLMMs) for inferential statistical analysis.

Table 2 presents the perceptual assimilation patterns of Mandarin sibilants to Cantonese categories by the Guangzhou participants, organized as a cross-linguistic confusion matrix across two distinct vocalic contexts (/an/ and /uan/). The rows list the 9 Mandarin target sibilants, systematically grouped by manner of articulation (unaspirated affricates, aspirated affricates, and fricatives), while the columns represent the 5 Cantonese response options. Within each cell, the upper value indicates the descriptive assimilation proportion (%), representing the selection frequency relative to the total trials for that target. The lower value in parentheses denotes the corresponding mean similarity rating scored on the 7-point Likert scale.

**Table 2:** Proportions (%) of Mandarin sibilants assimilation to Cantonese consonants by Guangzhou participants.

Rime	Cantonese response	Mandarin target sibilants								
		Unaspirated affricate			Aspirated affricate			Fricative		
		ts	tʂ	ʨ	tsʰ	tʂʰ	ʨʰ	s	ʂ	ɕ
an	tʃ	**84.2%** **(5.1)**	**92.1%** **(5.3)**	**84.2%** **(3.0)**			10.5% (1.5)			7.9% (1.7)
	tʃʰ	5.3% (5.5)	2.6% (3.0)	5.3% (4.5)	**100%** **(6.0)**	**97.4%** **(5.9)**	**73.7%** **(3.2)**			2.6% (1.0)
	ʃ	5.3% (5.5)		2.6% (5.0)		2.6% (7.0)	13.2% (4.0)	**100%** **(6.1)**	**100%** **(6.0)**	**89.5%** **(3.6)**
	t	5.3% (6.0)	5.3% (6.5)	7.9% (4.3)						
	tʰ						2.6% (1.0)			
uan	tʃ	**100%** **(4.7)**	**100%** **(4.4)**	**94.7%** **(4.7)**		2.6% (1.0)	2.6% (6.0)		5.3% (1.0)	2.6% (6.0)
	tʃʰ			5.3% (4.5)	**100%** **(4.7)**	**94.7%** **(5.1)**	**89.5%** **(4.7)**		5.3% (5.0)	
	ʃ					2.6% (6.0)	7.9% (4.3)	**100%** **(5.6)**	**89.5%** **(5.6)**	**97.4%** **(5.7)**
	t									
	tʰ									

Bold values indicate response proportions that are above chance level. Values in parentheses are mean similarity ratings on a 7-point scale (1 = least similar, 7 = most similar).

The table illustrates a distinct cross-linguistic mapping pattern: across both /an/ and /uan/ rimes, Guangzhou participants consistently assimilated Mandarin sibilants into Cantonese categories sharing the identical manner of articulation. Specifically, Mandarin affricates and fricatives were predominantly mapped onto their corresponding Cantonese manner blocks, with assimilation proportions frequently exceeding 84% and reaching up to 100% in multiple contexts. This robust alignment indicates high sensitivity to manner cues (e.g., affrication and aspiration), resulting in minimal cross-manner misjudgments (rarely exceeding 10%). Conversely, substantial perceptual overlap occurred within each manner block, demonstrating that listener confusion is primarily constrained to the place of articulation.

**Figure 2:**
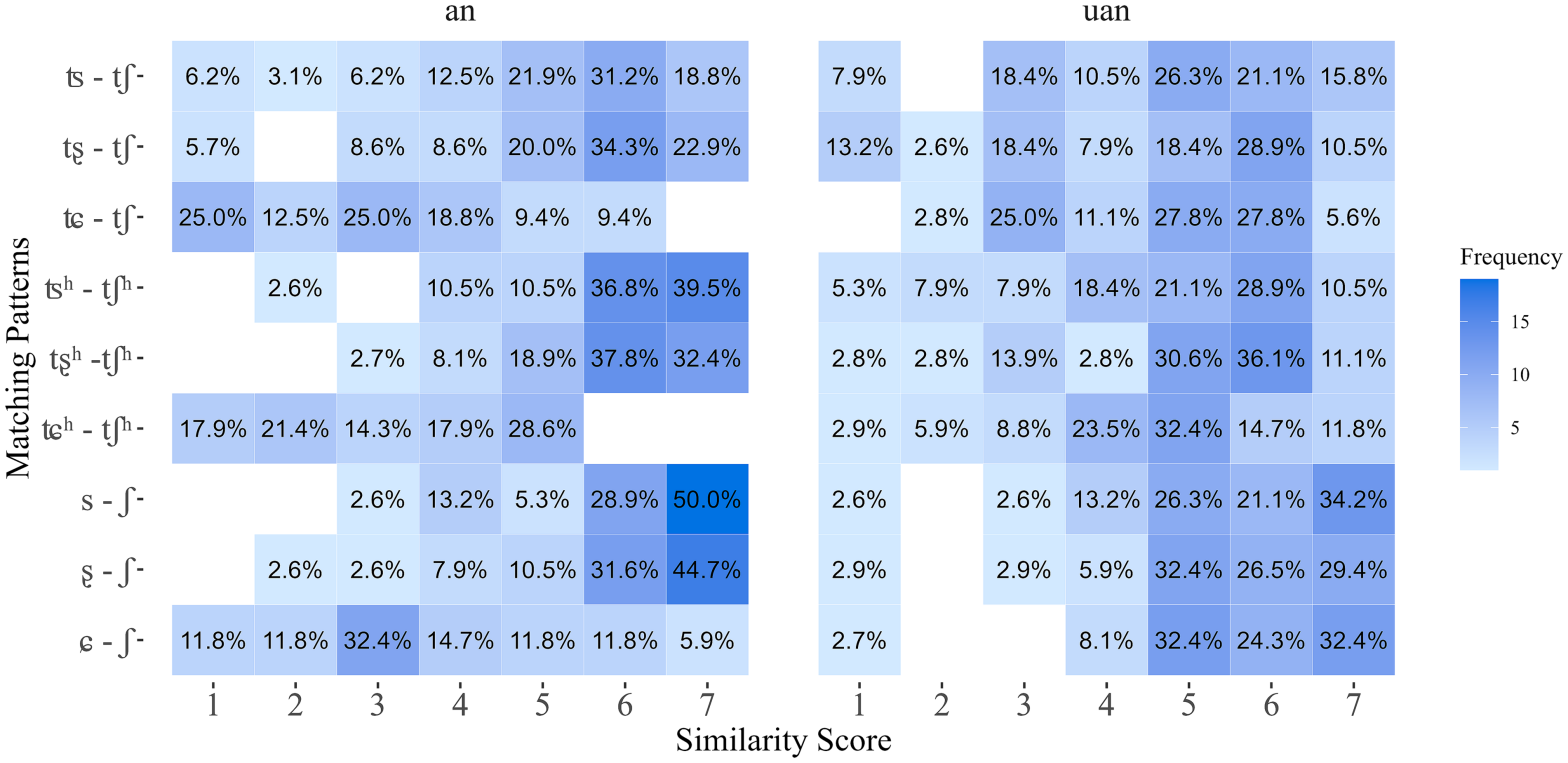
Perceived similarity ratings of sibilants by cantonese participants.

While the aforementioned data reflect preliminary trends, it is more critical to identify the nuanced differences in perceived similarity within the same assimilation patterns. To this end, we calculated the frequency distribution of similarity ratings (ranging from 1 to 7) and presented the results via heatmaps. Figure 2 displays the similarity ratings provided by Cantonese participants for sibilants in the two rime environments. The horizontal axis represents the similarity scores, while the vertical axis indicates the participant’s selections (i.e., the assimilation outcomes). The percentages within the figure indicate the frequency proportion of each similarity score within its corresponding matching group. For example, in the /an/ rime environment, for the [ts]-[tʃ] match, the proportions for ratings of 7, 6, and 5 were 18.8, 31.2, and 21.9%, respectively. Together, these exceed 70%, indicating that the majority of participants perceived a high degree of similarity between Mandarin [ts] and Cantonese [tʃ]. Cells remain blank where no corresponding ratings were recorded.

As illustrated in Figure 2, although Cantonese participants assimilated Mandarin sibilants into Cantonese categories based on their corresponding manners of articulation, they remained sensitive to the nuanced similarity differences across different places of articulation. This general trend, however, varied across rime environments and sibilant categories. Specifically, in the /an/ environment, participants perceived the two sets of Mandarin “apical” sibilants—namely, the dental-alveolars ([ts, tsʰ, s]) and the post-alveolars ([tʂ, tʂʰ, ʂ])—as being more similar to Cantonese laminal sibilants ([tʃ, tʃʰ, ʃ]) than to the alveolo-palatal series. This pattern was most pronounced in the fricative category, followed by aspirated affricates. Conversely, a different pattern emerged in the /uan/ rime environment, where the similarity scores between Mandarin alveolo-palatal sibilants and Cantonese laminal sibilants increased, exhibiting a performance more closely aligned with the other two series of articulation places. Additionally, within the affricate categories, the probability of the maximum rating (7) decreased, while the proportions in the mid-to-low score ranges increased.

Separate CLMMs were constructed for each manner of articulation (unaspirated affricates, aspirated affricates, and fricatives). To target the relevant perceptual contrasts, only trials in which the Mandarin target stimulus was assimilated to a Cantonese response category with the same manner of articulation were retained for the CLMMs. For each model, the dependent variable was the ordinal similarity rating (ranging from 1 to 7), with *Place of Articulation, Rime*, and their interaction included as fixed predictors, and *Participant* modeled as a random intercept. The significance of the fixed effects was evaluated via likelihood-ratio tests. The omnibus tests revealed that the statistical significance of the main effects varied across analysis categories. For *Place of Articulation*, a highly significant main effect was found across all three CLMMs (unaspirated: *χ^2^*(2) = 21.87, *p* < 0.001; aspirated: *χ^2^*(2) = 47.38, *p* < 0.001; fricatives: *χ^2^*(2) = 25.98, *p* < 0.001). By contrast, the main effect of *Rime* was only significant for aspirated affricates (*χ^2^*(1) = 6.19, *p* = 0.013), but failed to reach significance for both unaspirated affricates (*χ^2^*(1) = 0.03, *p* = 0.864) and fricatives (*χ^2^*(1) = 1.70, *p* = 0.193). Importantly, these main effects were qualified by a highly significant interaction between *Place of Articulation* and *Rime* across all three CLMMs (unaspirated affricates: *χ^2^*(2) = 33.41, *p* < 0.001; aspirated affricates: *χ^2^*(2) = 48.42, *p* < 0.001; fricatives: *χ^2^*(2) = 43.81, *p* < 0.001), indicating that the perceptual effect of the target place was robustly modulated by the vocalic environment. Notably, the pairwise comparisons across different places of articulation within each vocalic environment (ref. Table 3) corroborated the perceptual distribution illustrated in Figure 2. In the unrounded /an/ rime environment, a highly consistent pattern emerged across all three manners of articulation: no significant differences in similarity ratings were observed between the Mandarin dental-alveolar and post-alveolar series when mapped onto their respective Cantonese laminal counterparts (all *p*s > 0.05). Strikingly, however, both series received significantly higher similarity ratings than the alveolo-palatal series (all *p*s < 0.001). In contrast, in the rounded /uan/ rime environment, this place-conditioned asymmetry was no longer statistically evident. The similarity ratings exhibited no statistically significant differences across all pairwise comparisons among the three Mandarin places of articulation (all *p*s > 0.05), regardless of their manner of articulation.

**Table 3:** *Post-hoc* pairwise comparisons based on cumulative link models for similarity ratings.

Rime	Series	Contrast	Estimate	*SE*	df	*z.*ratio	*p* value
an	unaspirated affricate	(ts - tʃ) - (tʂ - tʃ)	−0.40	0.47	Inf	−0.84	0.68
		(ts - tʃ) - (tɕ - tʃ)	3.03	0.52	Inf	5.83	<0.0001
		(tʂ - tʃ) - (tɕ - tʃ)	3.43	0.53	Inf	6.51	<0.0001
	aspirated affricate	(tsʰ - tʃʰ) - (tʂʰ - tʃʰ)	0.11	0.46	Inf	0.24	0.97
		(tsʰ - tʃʰ) - (tɕʰ - tʃʰ)	4.74	0.58	Inf	8.17	<0.0001
		(tʂʰ - tʃʰ) - (tɕʰ - tʃʰ)	4.63	0.58	Inf	8.03	<0.0001
	fricative	(s - ʃ) - (ʂ - ʃ)	0.15	0.46	Inf	0.33	0.94
		(s - ʃ) - (ɕ - ʃ)	3.70	0.52	Inf	7.07	<0.0001
		(ʂ - ʃ) - (ɕ - ʃ)	3.55	0.52	Inf	6.86	<0.0001
uan	unaspirated affricate	(ts - tʃ) - (tʂ - tʃ)	0.43	0.43	Inf	1.01	0.57
		(ts - tʃ) - (tɕ - tʃ)	0.20	0.43	Inf	0.47	0.88
		(tʂ - tʃ) - (tɕ - tʃ)	−0.23	0.43	Inf	−0.54	0.85
	aspirated affricate	(tsʰ - tʃʰ) - (tʂʰ - tʃʰ)	−0.65	0.43	Inf	−1.49	0.30
		(tsʰ - tʃʰ) - (tɕʰ - tʃʰ)	0.25	0.44	Inf	0.56	0.84
		(tʂʰ - tʃʰ) - (tɕʰ - tʃʰ)	0.89	0.44	Inf	2.04	0.10
	fricative	(s - ʃ) - (ʂ - ʃ)	0.04	0.43	Inf	0.09	1.00
		(s - ʃ) - (ɕ - ʃ)	−0.16	0.42	Inf	-0.37	0.93
		(ʂ - ʃ) - (ɕ - ʃ)	−0.20	0.43	Inf	−0.46	0.89

## 5 Discussion

### 5.1 Perceptual assimilation patterns and contextual variation of sibilants

The primary objective of this study was to examine how native Cantonese speakers perceive Standard Mandarin sibilants within a refined, text-free experimental framework. Our results yielded two major phonological findings. First, participants demonstrated a robust sensitivity to manner of articulation and aspiration contrasts, consistently distinguishing between fricatives and affricates, as well as aspirated and unaspirated counterparts. Their assimilation patterns were therefore primarily differentiated by manner and aspiration, whereas confusion mainly occurred across places of articulation. This pattern aligns with the phonological architecture of the participants’ native language; since Cantonese possesses its own contrastive inventory of affricates and fricatives with aspiration distinctions, these distinctive features remain highly salient to Cantonese listeners, thereby preventing cross-manner perceptual mismatches.

Second, and more theoretically significant, our findings revealed that phonetic similarity is not static but varies dynamically as a function of the vocalic environment. Specifically, the rounding context (/uan/) significantly modulated the similarity ratings for Mandarin sibilants. In the /an/ environment, the two types of Mandarin apical sibilants were highly assimilated to the Cantonese laminal sibilant (with scores clustered between 5 and 7). However, in the /uan/ environment, the similarity ratings between the Mandarin alveolo-palatals and the Cantonese laminal sibilants increased markedly (with scores rising above 4, indicating a moderate-to-high similarity).

This perceptual shift mirrors long-standing dialectological observations. In Cantonese phonotactics, the laminal sibilants are permitted to co-occur with the high front rounded vowel [y], a distribution that highly correlates with the phonotactics of Mandarin alveolo-palatals, which are exclusively compatible with high front vowels. Dialectologists have noted that under the influence of such rounded vocalic environments, Cantonese sibilants historically exhibit distinct palatalization tendencies (Wong, 1941; Kao, 1971; Bauer and Benedict, 1997; Cheung, 2002; Yu, 2016; Chan, 2017). The subtle shift in our empirical data demonstrates that the participants were sensitive to fine-grained acoustic-phonetic variation and project it onto their cross-linguistic perceptual spaces.

### 5.2 Methodological validation and operational advantages of the refined PAT

To validate the efficacy and validity of the refined PAT paradigm, we evaluate its performance against three core benchmarks, all of which supported our initial hypotheses. First, the empirical data strongly align with previous dialectological accounts. Our results showed that Cantonese speakers frequently mapped different Mandarin places of articulation onto the same Cantonese category, confirming the consensus in L2 acquisition literature that distinguishing Mandarin places of articulation (e.g., dental-alveolars vs. post-alveolars) constitutes the most formidable challenge for Cantonese learners (Li et al., 1988; Zeng, 1996; Zong, 2000; He, 2001; Wu, 2011; Wang, 2014; Hu, 2016). Second, the paradigm demonstrated the sensitivity required to capture the context-conditioned phonetic variations discussed in Section 5.1. This finding underscores the capacity of our refined approach to bridge the gap between empirical behavioral data and theoretical dialectological descriptions. Third, the successful implementation of this paradigm across both affricate and fricative categories demonstrates its broad applicability across various sound classes. In phonological typology, consonants—particularly voiceless fricatives and affricates—exhibit lower perceptual independence than vowels, as their acoustic and perceptual characteristics are heavily conditioned by the subsequent vocalic environment. The robust sensitivity demonstrated in the current study suggests that our paradigm is highly generalizable to the investigation of other complex consonantal systems.

Beyond its methodological validity, the refined paradigm offers distinct design and operational advantages. Chief among these is a largely orthography-independent response format, which decouples speech perception from orthographic interference. This is particularly crucial for logographic writing systems like Chinese *Hanzi*, where characters cannot be decomposed into individual segments and their phonetic representations span beyond a single phoneme. Furthermore, by utilizing audible, syllable-based stimuli rather than isolated segments, the paradigm maximizes ecological validity. Psycholinguistic research indicates that the syllable, rather than the phoneme, serves as the primary phonological processing unit in Chinese (O’Seaghdha et al., 2010; Zhang and Wang, 2020). Forcin g logographic learners to map sounds onto isolated text or IPA symbols constitutes an unnatural metalinguistic task. Our syllable-bound is more consistent with the phonological processing mechanisms of non-alphabetic learners, making it uniquely suited for speech research across diverse Chinese dialects that are mutually unintelligible yet share a logographic script.

In terms of field execution, the refined PAT retains the classic advantage of a low task demand. Implemented via open-source behavioral software, it requires no specialized laboratory hardware, ensuring high practical utility. The procedural instructions are minimalist; naive participants without any phonetic notation training can easily complete the task by focusing solely on the initial consonant they hear. Procedural artifacts are further mitigated by our specific design features: implementing a fixed candidate order helps participants establish stable, internal cognitive representations of the response options. This layout familiarity not only expedites the experimental procedure but also minimizes cognitive fatigue.

### 5.3 Theoretical implications for speech acquisition models

Historically, the cross-linguistic similarity between Mandarin and Cantonese sibilants has been evaluated primarily through IPA symbol comparison. As noted by Flege (1987, 1992), IPA transcription serves as a primary, direct metric for evaluating L1–L2 phonetic distance. Under the SLM framework (Flege, 1995, 1997), this symbol-based taxonomy operates on a two-tiered logic: L2 sounds sharing identical IPA labels with L1 counterparts are further differentiated into “identical” or “similar” categories based on their acoustic nuances; conversely, L2 sounds transcribed with divergent IPA symbols are fundamentally treated as “new” sounds. However, this symbol-based taxonomy suffers from profound subjectivity. For instance, scholars like Lee and Wong (2020) classified Mandarin dental sibilants and Cantonese laminals as “similar,” whereas Wu and Su (2014) and Li (2018) classified them as “identical”—a discrepancy driven entirely by differing phonetic transcription conventions. Conflicting categorizations abound regarding retroflexes and alveolo-palatals as well, with different studies cross-labeling them as either “new” or “similar.” Ultimately, such symbol-dependent alignments overlook crucial allophonic variations conditioned by specific vocalic environments, thereby leading to conflicting predictions regarding L2 speech learning outcomes.

The empirical results obtained from our refined paradigm are capable of resolving these long-standing contradictions. While the traditional PAT paradigm already marked a pivotal conceptual shift by replacing subjective expert transcriptions with listener-centered perceptual data, its empirical implementation within the classical SLM framework (Flege, 1997) has conventionally operated on a discrete, nominal “identical–similar–new” tri-fold classification. Complementing this methodological evolution, our approach inherits the advantages of prior methodologies that combine classification tasks with scalar measurements; specifically, it integrates explicit categorical choice options with gradient interval ratings (ranging from 1 to 7) to move beyond these rigid categorical boundaries, offering a highly sensitive, continuous metric. This alignment directly supports the Revised Speech Learning Model (SLM-r; Flege et al., 2021), which conceptualizes the continuous magnitude of perceived phonetic distance—rather than a broad categorical label—as the critical determinant of whether a learner undergoes “category assimilation” or achieves “new category formation.” By applying this continuous approach to cross-linguistic similarity within a quantifiable perceptual space, this paradigm provides the empirical granularity necessary to more precisely model individual learning trajectories.

### 5.4 Limitations and future directions

Despite its extensive utility, several inherent limitations of the refined paradigm warrant caution. Methodologically, when measuring the cross-linguistic similarity of syllable-initial consonants, the subsequent vocalic environment must be strictly controlled. However, different languages exhibit distinct phonotactic constraints, making it occasionally difficult to construct perfectly matched cross-linguistic syllable pairs. For tonal languages like Chinese, the potential confounding effect of lexical tones must also be carefully isolated, adding substantial complexity to the baseline experimental design. Future researchers must invest more efforts into constructing appropriate minimal pairs or utilizing speech synthesis techniques to manipulate specific acoustic cues.

A specific empirical limitation of the present study concerns participant hearing screening. Although participants reported no history of hearing impairment or speech disorders and underwent a simplified hearing screening procedure before the experiment, laboratory-grade pure-tone audiometry was not conducted due to fieldwork constraints. Therefore, the possibility of mild or undetected high-frequency hearing loss cannot be fully excluded, particularly given the age range of the participants and the acoustic characteristics of fricatives and affricates. Future studies should incorporate rigorous clinical or laboratory-based audiometric screening to better control for this potential confound.

## Data Availability

The datasets presented in this study can be found in online repositories. The names of the repository/repositories and accession number(s) can be found at: https://osf.io/cwvmh/overview?view_only=e790748ff9824640a1bd1ea1574faeea.

## Footnotes

1 Wong (1941) only referred to the high front rounded vowel context, whereas Bauer and Benedict (1997) identified the rounding environment as encompassing all non-back rounded vowels.

2 The Mandarin /uan/ rime was realized as [uan] after dental-alveolar and post-alveolar sibilants, but as [yan] after alveolo-palatal sibilants, depending on the transcription convention adopted.
